# Perioperative celecoxib administration for pain management after total knee arthroplasty – A randomized, controlled study

**DOI:** 10.1186/1471-2474-9-77

**Published:** 2008-06-03

**Authors:** Yu-Min Huang, Chiu-Meng Wang, Chen-Ti Wang, Wei-Peng Lin, Lih-Ching Horng, Ching-Chuan Jiang

**Affiliations:** 1 Department of Orthopaedic Surgery, National Taiwan University Department of Orthopaedic Surgery, National Taiwan University Hospital, Taipei, Taiwan; 2 Department of Orthopaedic Surgery, Shuang Ho Hospital of Taipei Medical University, Taipei, Taiwan; 3 Department of Orthopaedics, Kinmen Hospital Department of Health, Kinmen, Taiwan; 4 Department of Nursing, College of Medicine, National Taiwan University & Hospital, Taipei, Taiwan

## Abstract

**Background:**

Non-steroidal anti-inflammatory drugs (NSAIDs) are recommended for multimodal postoperative pain management. We evaluated opioid-sparing effects and rehabilitative results after perioperative celecoxib administration for total knee arthroplasty.

**Methods:**

This was a prospective, randomized, observer-blind control study. Eighty patients that underwent total knee arthroplasty were randomized into two groups of 40 each. The study group received a single 400 mg dose of celecoxib, one hour before surgery, and 200 mg of celecoxib every 12 hours for five days, along with patient-controlled analgesic (PCA) morphine. The control group received only PCA morphine for postoperative pain management. Visual analog scale (VAS) pain scores, active range of motion (ROM), total opioid use and postoperative nausea/vomiting were analyzed.

**Results:**

Groups were comparable for age, pre-operative ROM, operation duration and intraoperative blood loss. Resting VAS pain scores improved significantly in the celecoxib group, compared with controls, at 48 hrs (2.13 ± 1.68 vs. 3.43 ± 1.50, p = 0.03) and 72 hrs (1.78 ± 1.66 vs. 3.17 ± 2.01, p = 0.02) after surgery. Active ROM also increased significantly in the patients that received celecoxib, especially in the first 72 hrs [40.8° ± 17.3° vs. 25.8° ± 11.5°, p = 0.01 (day 1); 60.7° ± 18.1° vs. 45.0° ± 17.3°, p = 0.004 (day 2); 77.7° ± 15.1° vs. 64.3° ± 16.9°, p = 0.004 (day 3)]. Opioid requirements decreased about 40% (p = 0.03) in the celecoxib group. Although patients suffering from post-operative nausea/vomiting decreased from 43% in control group to 28% in celecoxib group, this was not significant (p = 0.57). There were no differences in blood loss (intra- and postoperative) between the groups. Celecoxib resulted in no significant increase in the need for blood transfusions.

**Conclusion:**

Perioperative celecoxib significantly improved postoperative resting pain scores at 48 and 72 hrs, opioid consumption, and active ROM in the first three days after total knee arthroplasty, without increasing the risks of bleeding.

**Trial registration:**

Clinicaltrials.gov NCT00598234

## Background

Surgical trauma induces the synthesis of prostaglandins, which sensitize the peripheral nociceptors [1]. Non-steroidal anti-inflammatory drugs (NSAIDs) inhibit prostaglandin synthesis in the periphery and the spinal cord, therefore decreasing the post-operative hyperalgesic state [2]. NSAIDs have been shown to have opioid-sparing effects [3-6] and reduce postoperative nausea and vomiting (PONV) by 30% [7]. However, the analgesic effects of perioperative NSAIDs are still uncertain [8-10]. Some studies suggest that perioperative NSAIDs improve postoperative pain for ambulatory arthroscopic knee [11] or spinal fusion surgery [12]. However, few papers have discussed the effectiveness of perioperative NSAID in pain management after total knee arthroplasty (TKA) [13-15]. Rofecoxib has been the perioperative coxib in previous studies. However, in September 2004, it was withdrawn from the market due to its thromboembolic effects, particularly myocardial infarction.

Celecoxib is a selective cyclooxygenase (COX)-2 inhibitor and an effective analgesic for acute postoperative pain [16]. Although pre-operative non-selective NSAID use increases the risks of bleeding [10,17], celecoxib (1200 mg daily) has no effects on serum thromboxane or platelet functions [16]. Celecoxib (400 mg) also has similar analgesic effects in comparison with conventional non-selective NSAID [4]. To achieve less postoperative pain and better rehabilitation after TKA surgery, especially in the first week, prescription of oral celecoxib preemptively for pain management of TKA patients is reasonable. Although previous studies have evaluated the analgesic efficacy of rofecoxib, few studies to date have evaluated the efficacy of celecoxib for TKA. In this study, we hypothesized that celecoxib provides better efficacy than the use of patient-controlled analgesic (PCA) morphine, which is currently the standard therapy in our institute. We aimed to compare the difference in the pain scores at rest and ambulation, along with range of motion (ROM), morphine-sparing effects, PONV, and perioperative blood loss between patients receiving celecoxib treatment and patients receiving PCA morphine treatment after TKA surgeries.

## Methods

This study was performed (from September 2006 to March 2007) after institutional IRB approval. All TKA surgeries were performed by one surgeon (Ching-Chuan Jiang). Under a randomized, prospective, observer-blind study design, subjects were sorted by random numbers into two groups. Inclusion criteria for this study were primary osteoarthritis and an age over sixty. Exclusion criteria for this study were rheumatoid arthritis, end-stage renal disease (complete kidney dysfunction requiring dialysis or kidney transplantation), previous cerebral vascular accident history, peptic ulcers, recent myocardial infarction (within 1 year), and allergy to sulf, NSAIDs, or morphine. The National Taiwan University Hospital and National Institutes of Health have approved the study and all participants will provide written informed consent.

The study group (n = 40) received 400 mg oral celecoxib at about 1 hr prior to surgery, and 200 mg every 12 hrs, along with PCA morphine, over the first five post-operative days. The control group (n = 40) received PCA morphine over the same postoperative period.

All patients had spinal anesthesia and hemovac drain tubes inserted for postoperative blood loss evaluation. Patient data included gender, age, range of motion (ROM), pain scores, blood loss and procedure duration. Pain scores were measured using a visual analog scale (VAS), with 0 indicating no pain and 10 indicating the worst imaginable pain [18]. Pain scores were checked by the same observer at rest at 6, 12, 24, 48 & 72 hrs and 7 days after TKA surgery. We encouraged patients to ambulate 24 hrs after TKA. Pain scores at ambulation were also analyzed. We measured the range of motion before ambulation by using a goniometer. Patients were supine on the bed and we identified bony landmarks, including the greater trochanter, lateral femoral condyle, and the lateral mallelous, to facilitate goniometer placement. All ranges of motion were measured preoperatively and postoperatively by the same study nurse (Lih-Ching Horng). The CPM machine was used twice a day in all patients. PCA morphine dose and PONV occurrence were also recorded. All NSAIDs were discontinued at seven days after surgery. All patients received a patient-controlled analgesia (PCA) pump after surgery. Morphine (1 mg/mL) was given intravenously with increases of 2 mL and a lockout interval of 10 minutes. Additionaly, thromboembolic prophlaxis (oral aspirin 100 mg) was given for seven postoperative days in both groups.

The primary endpoint was VAS pain reduction through perioperative celecoxib administration. Group sample sizes of 40 and 40 can achieve 85% power in the detection of a difference of one point on a 10-point VAS scale, which is equivalent to the minimal clinical significant difference on the VAS scale [19], in a design with four repeated measurements when the standard deviation is 1.8 points. Group sample sizes of 40 and 40 can achieve 91% power in the detection of a difference of one point for a 10-point VAS scale in a design with six repeated measurements, when the standard deviation is 1.8 points. Secondary endpoints include ROM, morphine-sparing effects, postoperative nausea, vomiting and blood loss.

Patient demographics were analyzed by ANOVA or Chi-squared test. Postoperative pain scores, postoperative ROM, morphine doses, blood loss, and PONV rates were analyzed by Mann-Whitney test. Group differences in pain intensity and ROM were also analyzed by repeated measures ANOVA test. Significance was defined as p < 0.05.

## Results

From Sep 2006 to March 2007, ninety-seven patients that fulfilled inclusion criteria received elective TKA surgery by the senior author (Ching-Chuan Jiang) at the author's institute. Fifteen patients were excluded (four with RA, three with ESRD, six with previous CVA, and two with a history of peptic ulcers). Two patients suffered from epigastralgia, without tarry stool, during the study and were also excluded, leaving a total of eighty patients. They were randomized into two groups. There were no significant differences between them in age, preoperative ROM, preoperative pain scores, operation duration, and intraoperative blood loss (Table 1).

**Table 1:** Patient demographics and surgical data. There were no significant differences between these groups.

**Group**	**Age (yr)**	**Pre-OP ROM (degrees)**	**Pre-OP VAS**	**Surgery Duration (min)**	**Tourniquet Duration (min)**	**Intra-operative blood loss (ml)**
Control	70 ± 7	118 ± 17	4.7 ± 1.9	75 ± 17	45 ± 13	177 ± 54
Celecoxib	70 ± 7	116 ± 18	5.1 ± 1.7	77 ± 17	46 ± 17	181 ± 63

### VAS pain scores

**Figure 1:**
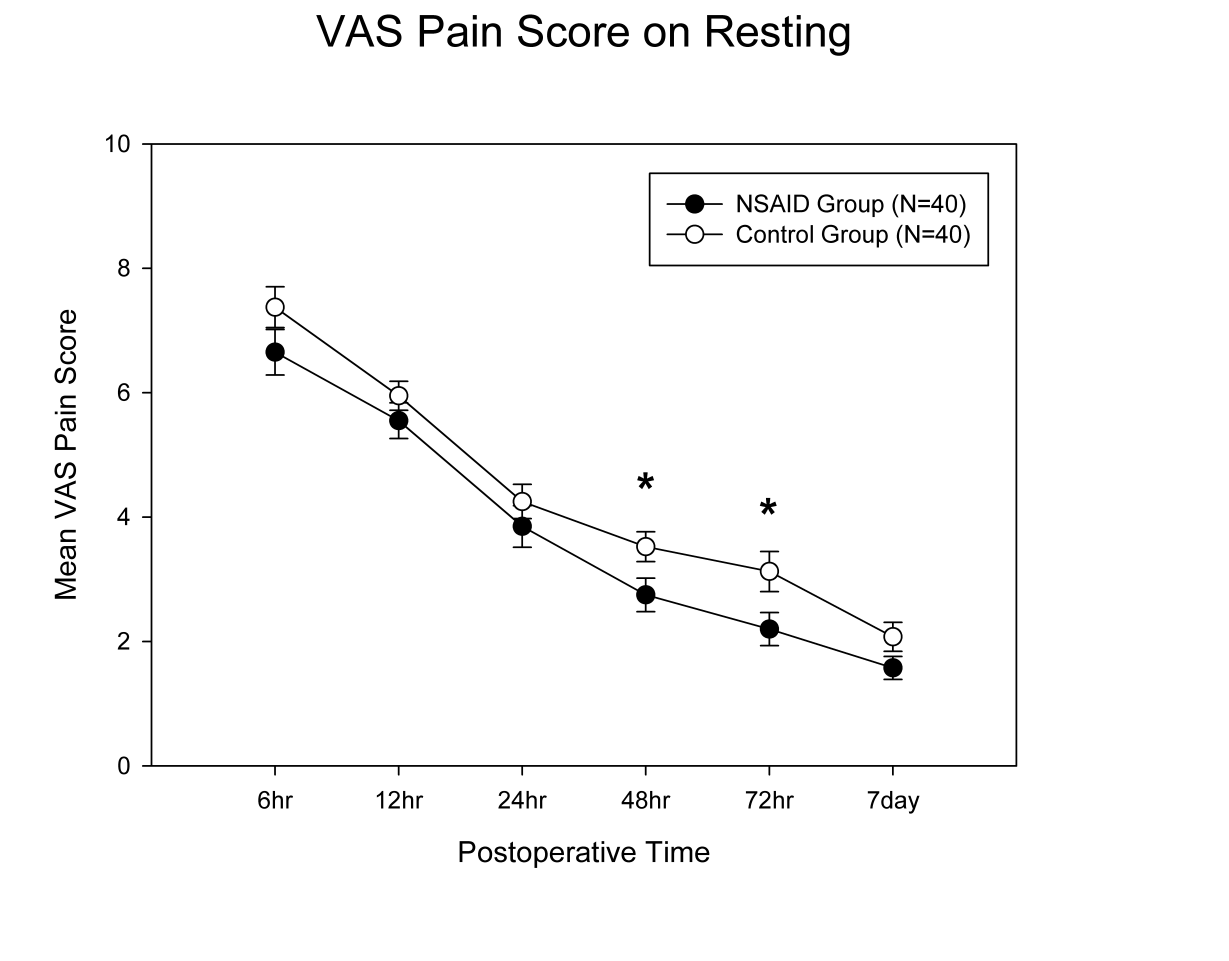
**Pain scores at rest**. The celecoxib group had significantly less pain on Days 2 and 3. *: p value < 0.05, and error bar indicates standard deviation.

**Figure 2:**
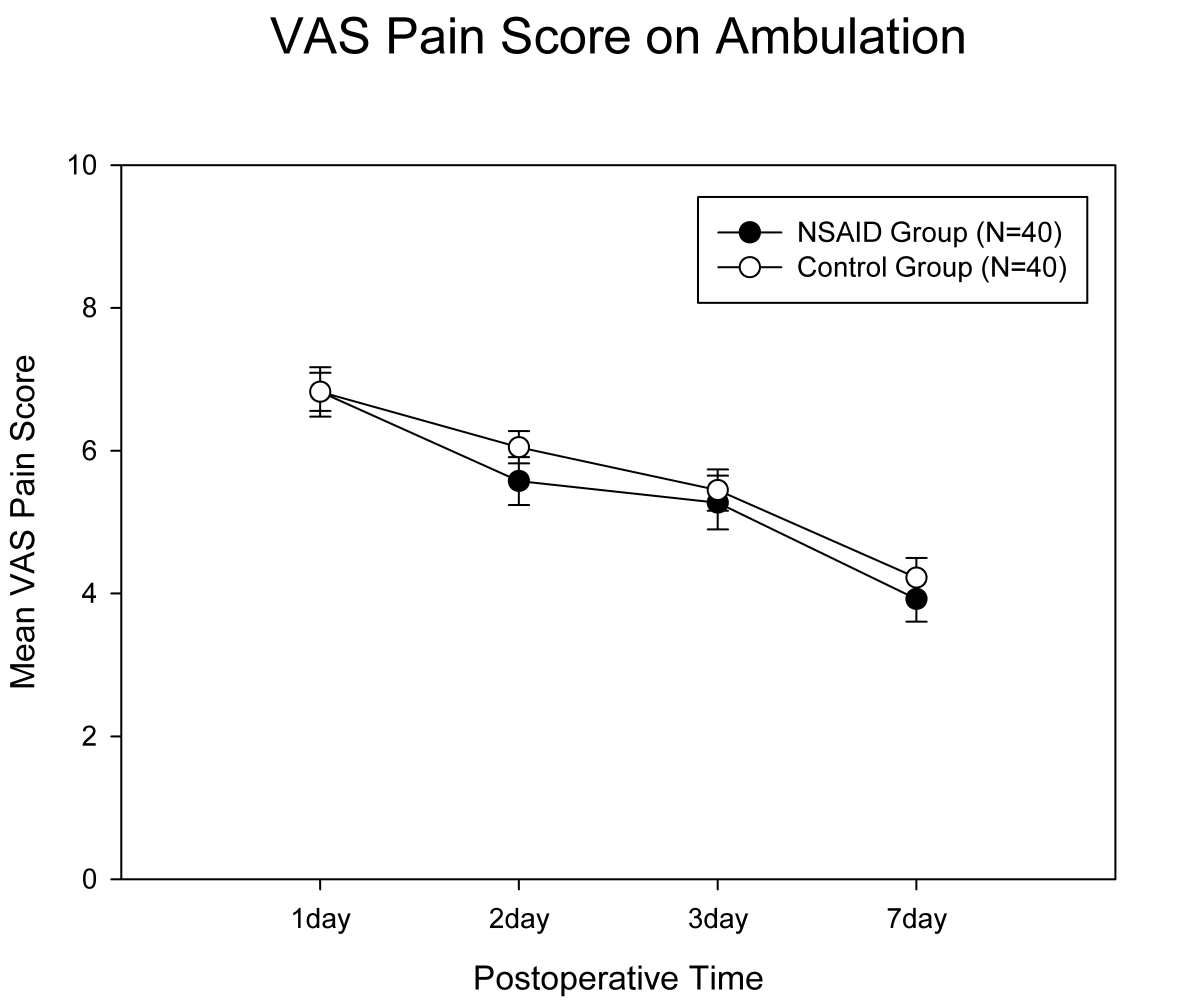
**Pain scores at ambulation**. There were no differences between the two groups. Error bar indicates standard deviation.

The celecoxib group showed less postoperative VAS pain at rest than the control group at 48 hrs (p = 0.03) and 72 hrs (p = 0.02) after TKA surgery (Fig 1). The celecoxib group had reduced postoperative pain at ambulation, but did not reach significant difference when compared with controls (Fig 2). Repeated measures ANOVA showed that the celecoxib group had significant VAS pain score reduction over the control group at rest (p = 0.023) but not during ambulation (p = 0.51).

### Range of motion

**Figure 3:**
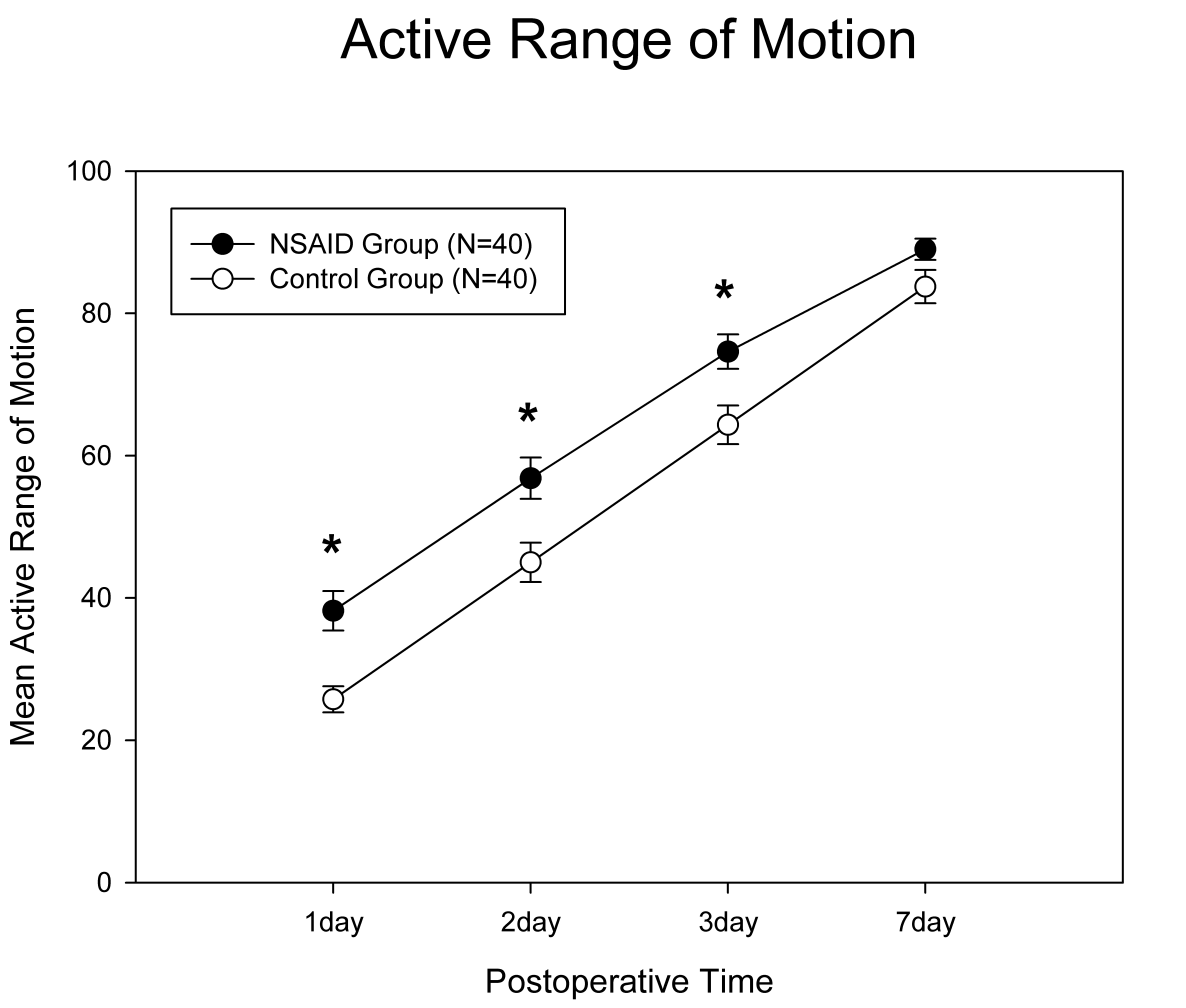
**Results for the postoperative range of motion**. The celecoxib group had significantly better active ROM during the first 3 days. *: p value < 0.05, and error bar indicates standard deviation.

Celecoxib significantly improved postoperative knee ROM, especially during the first three days (Day1: p = 0.01; Day 2: p = 0.004; Day 3: p = 0.004) (Fig 3). This group also had increased active ROM of 12–15 degrees in comparison with the control group. Repeated measures ANOVA showed that the celecoxib group also had significantly better postoperative ROM improvement than the control group (p = 0.0009).

### Morphine-sparing effects and postoperative nausea and vomiting

**Figure 4:**
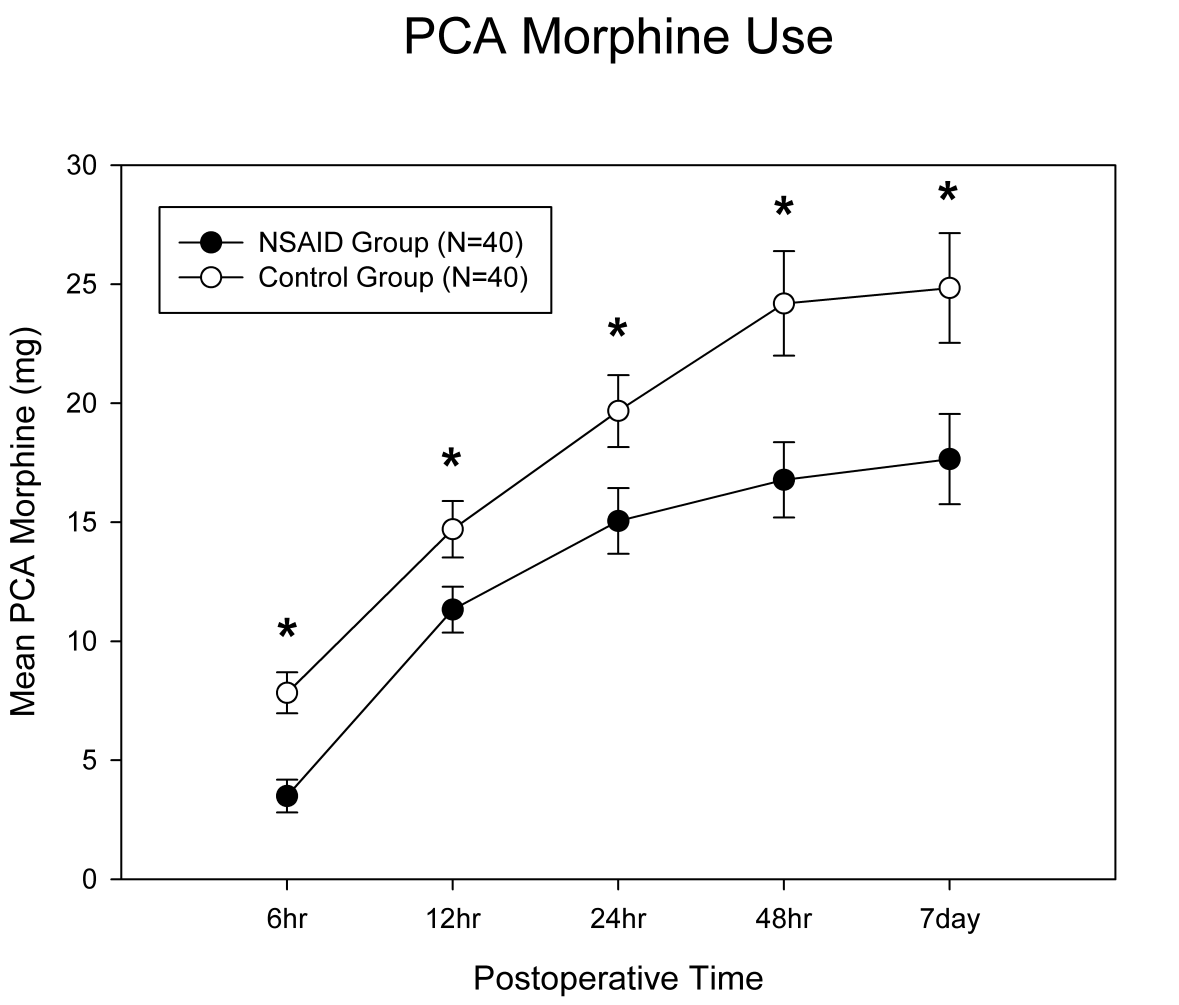
**PCA morphine use**. Mean PCA morphine use was significantly lower at each measurement for the celecoxib group. *: p value < 0.05, and error bar indicates standard deviation.

The celecoxib group had significantly less demand for PCA morphine usage (Fig 4). PCA usage during the first 24 hours was significantly lower (15.1 ± 8.7 mg vs 19.7 ± 9.6 mg; p = 0.03). Total PCA morphine usages in the celecoxib group and controls were 17.6 ± 11.9 and 24.6 ± 14.6 mg, respectively (p = 0.03). The celecoxib group used about 40% less morphine. Postoperative nausea and vomiting were at 28% and 43% in the celecoxib group and controls, respectively. However, this did not reach statistical significance (p = 0.57).

### Bleeding

There were no significant differences in intraoperative blood loss (181 vs 177 ml), postoperative blood loss through Hemovac drain tubes (544 vs 511 ml), and postoperative blood transfusions (3.85 vs 3.93 units) between the celecoxib group and controls.

## Discussion

In our study, we found that perioperative administration of celecoxib, a selective COX-2 inhibitor, reduced postoperative VAS pain scores at rest and decreased morphine usage while providing better ROM rehabilitation results. Perioperative administration of celecoxib did not increase perioperative bleeding or blood transfusion amounts.

Coxibs are effective analgesics for post-operative pain [20-22]. To the best of our knowledge, the literature lacks a review of perioperative celecoxib for pain management in TKA patients. Perioperative coxibs have been shown to reduce postoperative pain and analgesic consumption while enhancing patient satisfaction [6]. In 2000, Reuben and Connelly studied perioperative use of celecoxib (200 mg) and rofecoxib (50 mg) for spinal fusion surgery [12]. They concluded that both celecoxib and rofecoxib decrease morphine consumption, but that celecoxib's effects are limited. In their follow-up study, preoperative celecoxib 400 mg showed better morphine-sparing effects than a single 200 mg preoperative dose of celecoxib [23]. Therefore, we suggest that 400 mg of celecoxib is more optimal than 200 mg in controlling acute postoperative pain [4,6]. In our study, although VAS scores were significantly improved at 48 and 72 hrs at rest, and repeated measures ANOVA test also indicated that the celecoxib group had significantly lower pain scores at rest (p = 0.023), there was no significant difference between the two groups in pain scores at ambulation. We also demonstrate that perioperative celecoxib improves postoperative ROM results. The mean knee ROM was 92 degrees in the celecoxib group and 83.8 degrees in the control group, seven days after surgery.

NSAIDs are known to decrease opioid consumption by 30–50% [6,7,24]. In our study, perioperative celecoxib significantly lowered it by 40%. However, the effects of perioperative coxibs on opioid-related side effects are still uncertain [6]. This study also showed reduced postoperative nausea and vomiting (28% vs. 43%). However, there were no differences in PONV incidence. Further evaluation, using a larger sample, is called for.

Previous studies have shown that conventional preoperative non-selective NSAIDs increase bleeding risks [17,25]. Conventional non-selective NSAIDs reversibly inhibit COX and interfere with platelet functions, while selective COX-2 inhibitors have less antiplatelet effects than conventional non-selective NSAIDs [26,27]. In this study, perioperative administration of 400 mg of celecoxib had no significant effects on blood loss, thus confirming indirectly that celecoxib has little effect on serum thromboxane or platelet functions [16]. The analgesic effects of aspirin may confuse the benefits of celecoxib, even though both groups are allowed to have aspirin in our study, and use of aspirin does not prevent the thromboembolic adverse effects of coxibs.

Selective COX-2 inhibitors may be a better choice for multimodal analgesia than conventional non-selective NSAID. Selective COX-2 inhibitors may have better gastrointestinal tolerance and less risk for cardiovascular events. It's suggested that selective COX-2 inhibitors have less gastrointestinal toxicity than conventional non-selective NSAIDs [28,29]. It's suggested that selective COX-2 inhibitors increase the risks of cardiovascular events, myocardial infarction, stroke and heart failure [29,30], too. White et al. also demonstrated no significant increase in CV risks with celecoxib in comparison to placebo or non-selective NSAIDs [31]. Moore et al. even demonstrated that celecoxib had less gastrointestinal and cardiovascular risk than conventional non-selective NSAIDs and all other coxibs [32]. However, there might not be any difference in the coxibs and conventional non-selective NSAIDs with respect to serious vascular events [33]. Selective COX-2 inhibitors may cause less bleeding than non-selective NSAID, because coxibs do not interfere the normal mechanisms of platelet aggregation and hemostasis, whereas non-selective NSAID produces significant reductions in platelet aggregation and serum thromboxane B_2 _[34]. It has been shown that prior exposure of non-selective NSAID such as ibuprofen in THR surgery significant increases bleeding [10]. Therefore, we suggest that selective COX-2 is optimal for multimodal analgesia.

One limitation of this study is the lack of a true placebo group. Placebo response has been observed in up to 30% of patients that undergo surgery. However, placebo usage may present ethical concerns in the postoperative care of TKA surgery; we therefore adopted an active-control trial design that compared celebrex treatment to the standard therapy (PCA morphine) at our institute. Another limitation was the limited research period. We recorded data for only the first seven days. Although we demonstrated that celecoxib had better rehabilitative results in the first week than PCA morphine, long-term efficacy is still in question. There may be many reasons for the resting VAS scores to not have reached significance until 48 hours after surgery. One possibility is that the true difference does exist and that a larger sample size is needed to draw out the difference, despite that we had shown that the sample size in our study had adequate power.

## Conclusion

Perioperative celecoxib, combined with opioids, is an effective and safe regimen for pain control in TKA patients. Perioperative celecoxib can significantly decrease postoperative pain scores at rest and opioid use, while improving postoperative ROM results for TKA surgeries. Considering the benefits, we suggest celecoxib for multimodal analgesia.

## Competing interests

The authors declare that they have no competing interests.

## Authors' contributions

Y–MH, C–MW, W–PL, and C–CJ were responsible for the design of this study. L–CH was the study evaluator and recorder. C–TW provided analytical support. Y–MH, C–TW and C–CJ prepared the writing of the manuscript. All authors read and approved the final manuscript.

## Pre-publication history

The pre-publication history for this paper can be accessed here:


